# Graphene Integrated Hydrogels Based Biomaterials in Photothermal Biomedicine

**DOI:** 10.3390/nano11040906

**Published:** 2021-04-02

**Authors:** Le Minh Tu Phan, Thuy Anh Thu Vo, Thi Xoan Hoang, Sungbo Cho

**Affiliations:** 1 Department of Electronic Engineering, Gachon University, Seongnam-si 13120, Korea; 2 School of Medicine and Pharmacy, The University of Danang, Danang 550000, Vietnam; 3 Department of Life Science, Gachon University, Seongnam-si 13120, Korea; vtathu0612@gmail.com (T.A.T.V.); xoanht89@gmail.com (T.X.H.); 4 Department of Health Sciences and Technology, GAIHST, Gachon University, Incheon 21999, Korea

**Keywords:** graphene integrated hydrogel, photothermal biomedicine, photothermal therapy, photothermal antibacterial, thermal-triggered drug delivery, bone regeneration

## Abstract

Recently, photothermal therapy (PTT) has emerged as one of the most promising biomedical strategies for different areas in the biomedical field owing to its superior advantages, such as being noninvasive, target-specific and having fewer side effects. Graphene-based hydrogels (GGels), which have excellent mechanical and optical properties, high light-to-heat conversion efficiency and good biocompatibility, have been intensively exploited as potential photothermal conversion materials. This comprehensive review summarizes the current development of graphene-integrated hydrogel composites and their application in photothermal biomedicine. The latest advances in the synthesis strategies, unique properties and potential applications of photothermal-responsive GGel nanocomposites in biomedical fields are introduced in detail. This review aims to provide a better understanding of the current progress in GGel material fabrication, photothermal properties and potential PTT-based biomedical applications, thereby aiding in more research efforts to facilitate the further advancement of photothermal biomedicine.

## 1. Introduction

Photothermal therapy (PTT) has emerged in the last few decades as a potential medical tool in the area of various diseases and medical therapy, such as cancer therapy [1,2], bacterial infection [3], bone regeneration and tissue engineering [4] and drug delivery [5]. PTT is one of the main types of phototherapeutic methods for disease treatment because of its localized ablation of the tissue of interest and minimal heating damage to normal tissues near the targeted tissues. The core principle of PTT is the application of external near-infrared (NIR) light, which is absorbed by highly efficient photothermal agents (PTAs) accumulated noninvasively within the targeted tissues to convert absorbed light into thermal energy, thereby increasing the kinetic energy to provide local overheating. Because the hypothermia effect occurs only in the presence of PTAs in the targeted tissues, PTT offers highly efficient and unique methods for disease treatment owing to its precise spatial-temporal effect, high sensitivity, high pharmacokinetics, few side effects, ease of manipulation, low invasive burden, speed and efficacy of treatment and low cost [6,7]. The primary prerequisite for an effective PTT is the efficient delivery of PTAs to the tissue of interest and to precisely direct the external light into the tissues where PTAs are located. To meet this requirement, the dominant strategy has been to create highly efficient and multifunctional nanomaterials as PTAs with sufficient photothermal conversion efficacy and high biocompatibility [8,9,10]. PTAs with high light absorption capacity, high photostability, minimal cytotoxicity and good biocompatibility are more favorable. It is also important to note that the PTA components, structures and size remarkably affect the pharmacokinetics of PTT, including drug absorption, distribution, metabolism and release [11,12]. Hence, highly integrated nanomaterials are crucial for enhancing the optical properties, particle stability and biocompatibility, while reducing undesirable effects or toxicity, especially for drug delivery and tumor therapy.

To date, a variety of NIR-responsive nanomaterials, including both organic [13,14] and inorganic agents [15,16] have been explored for PTA preparation. Among them, biomaterials with adequate biocompatibility are highly preferred. Hydrogels that possess the obligatory characteristics for biomedical applications, such as good biocompatibility and low toxicity, have been envisioned as a new promising class of PTAs [17,18]. A hydrogel is a group of polymeric materials that can form a three-dimensional (3D) network of hydrophilic soft polymers with several unique features, including hydrophilicity, viscosity, elasticity, high water content and tunable stiffness, making them able to be fashioned into specific level through manipulation with different crosslinker types [19,20]. Other excellent characteristics of hydrogels include their biodegradability [21] and ability to mimic the compositions and physicochemical properties of the natural extracellular matrix [22], making hydrogels a highly biocompatible material with negligible cytotoxicity [23]. Owing to these unique properties, significant research attention has been given to hydrogels in recent years for many applications in the field of biomedicine, such as tissue engineering [24], drug carriers [25], anticancer [26] and antibacterial therapies [27] and biosensors [28,29]. Notably, hydrogel-based systems have shown distinct advantages in PTT and intensive studies have been conducted to fabricate hydrogel-modified composites to enhance the efficacy of photothermal therapeutics [30,31]. Nevertheless, a major limitation of hydrogels is the lack of mechanical strength, making it a great challenge for therapeutic applications. One innovative strategy to overcome this issue is to incorporate other materials into hydrogels to precisely mimic the extracellular matrix and improve the composite stiffness [32]. Among the different nanomaterials, graphene and graphene-based nanocomposites have been proposed as a new promising class of photothermal materials and the incorporation of graphene into hydrogel networks has significantly improved the capacity of hydrogels owing to their superior mechanical, electrical and optical properties [33].

Graphene and its chemical derivatives graphene oxide (GO) and reduced GO (rGO) are two-dimensional carbon single layers that possess abundant functional groups on their surface (carboxyl and hydroxyl groups), novel physical properties (photothermal properties, photoluminescent properties and large specific surface area) and good biocompatibility [34]. Owing to these superior properties, graphene and graphene derivatives have sparked great attention for different applications in the field of biosensors, drug delivery and bioimaging [35,36]. Among different types of graphene, GO is a useful material for photothermal application and is commonly used as a basic building material for the fabrication of other graphene-based nanomaterials. GO nanosheets have been used as effective building blocks for improving the chemical and physical properties of new integrated nanocomposites, such as light absorption, thermal and electrical conductivity and flexibility [37]. Furthermore, graphene possesses strong optical absorption in the NIR regions [38], making it a promising candidate for photothermal applications compared to existing conventional photothermal materials. Owing to the aforementioned features of graphene and hydrogel, there have been increasing studies to explore graphene-based hydrogel (GGel) nanocomposites for NIR-mediated PTT. The existing GGels have exhibited improved physical, chemical and biological properties, making them great candidates as potential PTAs [39,40].

**Figure 1:**
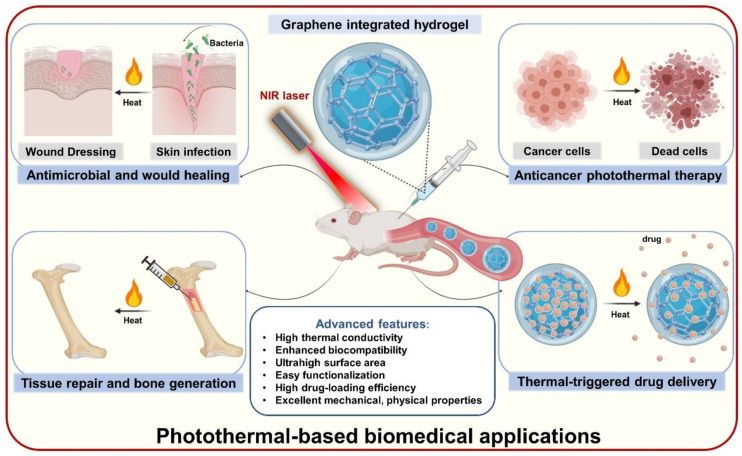
General illustration of advanced features and applications of graphene-based hydrogels as promising photothermal agents in diverse PTT-based biomedical fields, including anticancer photothermal therapy, thermal-triggered drug delivery, antimicrobial activity, wound healing acceleration, tissue repair and bone generation.

This review aims to introduce the current state-of-the-art in the development of innovative graphene-based hydrogels for PTT-based biomedical applications, as shown in Figure 1. The most up-to-date nanocomposites based on the integration of graphene and hydrogel, including their synthesis methods and thermal properties, will be introduced in detail while highlighting their recent and potential application in the field of PTT-based biomedicine.

## 2. Fabrication of Graphene-Integrated Hydrogels and Their Thermal Property

### 2.1. Fabrication of Graphene-Integrated Hydrogels

For the construction of graphene integrated hydrogel, graphene components are necessary to be synthesized first. GO sheets were fabricated from natural or high-purity graphite by using ultrasonication in water with several adjustments [41,42,43,44]. The incorporation of graphene into the polymer matrix exhibited outstanding performance, which not only facilitates three-dimensional handling, but can also considerably improve the mechanical strength of the hydrogel. For instance, only 3 wt% graphene complement in hydrogels could ameliorate the tensile strength by over 200% and the dispersion of graphene sheets in polymers was also increased through interfering with the strong π-π interactions between graphene sheets [45,46]. The principal hydrogel preparatory processes were employed to lay the foundation of graphene-incorporated polymer hydrogels with graphene or graphene derivatives. Currently, various methods have been investigated to generate graphene-incorporated hydrogels via inherent characteristic modifications by combining mixing and dispersing, freezing and thawing, shaking and sonication, or polymerization, providing valuable additives, including cross-linkers and ions.

#### 2.1.1. Physically Cross-Linked Fabrication

For graphene/hydrogel incorporation, mixing graphene dispersion is the simplest approach and is widely applied in numerous studies. Meanwhile, the procedures are proceeded by graphene/graphene oxide dissolution in water and this mixture is blended with diverse kinds of solvents, such as poly(vinyl alcohol) (PVA) [47,48,49], chitosan (CS) solution [50,51,52] and poly(acrylic acid) (PAA) [41], followed by more graphene sheet dispersion through sonication. Afterward, the mixtures are heated at an appropriate temperature to evaporate and are subjected to graphene-containing hydrogel formation. Typically, in the study by Rodrigues et al., CS and PAA were dissolved in HCl and mixed with a dispersing-GO solution, then stirred for 20 h and dropped onto NaOH to form hydrogel beads immediately [52].

**Figure 2:**
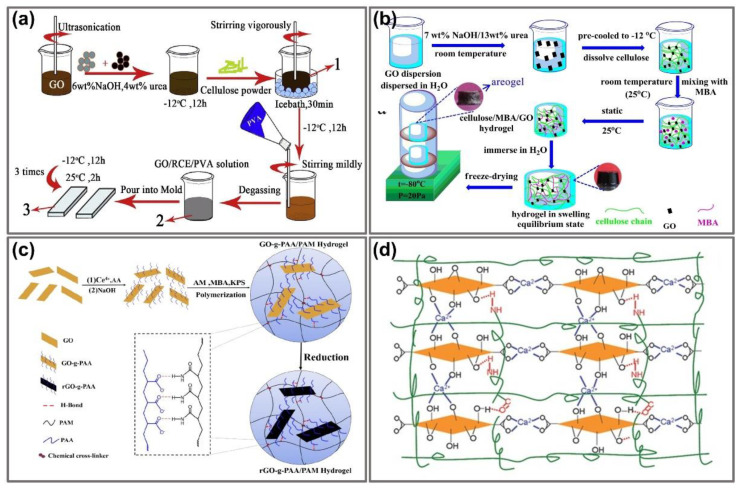
Graphene-immobilizing hydrogel preparation process. (**a**) Fabrication of GO–RCE/PVA ternary hydrogels by performing the freeze/thaw protocol. Adapted with permission from [53]. (**b**) Synthesis of cellulose/MBA/GO hydrogels by addition of MBA as a cross-linker. Adapted with permission from [66]. (**c**) Fabrication of GGels by in situ reduction of PAA grafted GO in the PAM matrix. Adapted with permission from [75]. (**d**) Schematic models of the GO/PAM hydrogel including a Ca^2+^ coordination-induced GO network (blue), chemically cross-linked PAM network (green) and hydrogen bonds between GO and PAM (red). Adapted with permission from [78].

Furthermore, hydrogel composite preparation is conducted by another simple method that repeats freeze-thaw cycles. Similarly, in the first step, a water-soluble synthetic polymer, such as PVA [42,43,49,53,54], PVA/polyethylene glycol (PEG) [55] and PVA/rigid polyaniline (PANI) [56], were prepared into a homogeneous solution and thoroughly blended with GO solution. Afterward, the mixture was poured into a container such as a plastic box or a mold and then the hydrogel composite was created by exposure to cooling and de-icing for several cycles. During these processes, both crystallization and separation are responsible for the hydrogel formation and mechanical property alterations [56]. For example, Xie et al. obtained regenerated cellulose (RCE)/PVA/GO hydrogels by performing freeze/thaw cycles. In particular, the PVA solution was mixed with a prepared-GO/RCE compound solution using ultrasonic stirring and cast into plastic molds. The freeze/thaw protocol was repeated three times, with a 12 h freeze at −20 °C and a 2 h thaw at ambient temperature [53] (Figure 2a).

After blending the GO solution with an aqueous polymer, such as amidoxime solution [57], PVA solution [58,59,60] and chitosan [61], graphene-based hydrogels were obtained through a simple combination of violent shaking and sonication. Chen et al. employed this approach to synthesize GO-CS hydrogels in which the GO suspension was blended with CS solution and, eventually, the hydrogel was formed after 10 s of violent vibration, followed by 10 min of sonication [61]. 

Among the diverse materials mentioned above, PVA is a biocompatible synthetic polymer that is widely employed because of its inherent properties, including good elasticity and the potential to synthesize the physical cross-linking of the hydrogel [62]. The overall performance of the conventional method is simple, inexpensive and easy to fabricate; however, the crosslinking dispersion of the GO is irregular, leading to destitute mechanical properties [63].

#### 2.1.2. Addition of Chemical Cross-Linkers

The 3D network hydrogels are easily generated by the self-assembled ability of GO sheets in a suitable solvent upon the attachment of a proper chemical cross-linker. In comparison with others, this method was regarded as a “green” process with lots of advantages, such as being simple and non-expensive, especially in the absence of any catalyst and forming equipment. This “one-step” method requires diverse crosslinkers including epichlorohydrin [64,65], N,N′-methylene bisacrylamide (MBA) [66] and 1,4-butanediol diglycidyl ether [67]. Typically, the cellulose/MBA/GO hydrogel was fabricated by dispersing GO into the prepared cellulose solution [66]. Firstly, the cellulose solution was obtained by dissolving cellulose in urea and NaOH and stirring at 0–4 °C. Subsequently, the MBA powder was added to the above solution under constant stirring. Finally, the cellulose/MBA/GO hydrogel was fabricated after seating for 12 h in a mold (Figure 2b).

#### 2.1.3. In Situ Polymerization

Polymerization consists of certain localized and propagated reaction chains to convert monomers into polymers and exhibits considerable advantages, including being quick and having low energy cost [68]. Therefore, using this method, various GO/hydrogel composites were prepared through the in situ polymerization of aniline [69], pyrrole [70], iron acetate [46], acrylic acid [63,71], polyacrylamide (PAM) [72], polymethyl methacrylate (PMMA) [73] and polyacrylamide-co-poly(acrylic acid) (PAM-co-PAA) [68,74,75] in GO dispersions with suitable peroxide initiators attached by heat or radiation [76]. The most used initiators were investigated, including ammonia persulfate [63,70,71,74] and potassium persulfate [68,75]. For instance, Zhang and colleagues successfully fabricated graphene oxide/polyacrylamide composite hydrogels using in situ polymerization of a mixture of monomer acrylamide and GO with the incidence of co-crosslinker N,N-methylene bisacrylamide [74]. Subsequently, the substance was poured into the cylinder mold and the reaction was conducted at 65 °C for 4 h. The incorporation of GO nanosheets and PAM macromolecules by a radical reaction in this method could trigger mechanical properties including compressive strength, bending angle and electric response. Furthermore, to ameliorate the homogeneous diffusion and interfacial interaction in the hydrogel network, Li and coworkers employed rGO instead of GO to assemble into polymer composites through a three-step method [75]. For this purpose, the process was established in the following order: preparation of PAA-grafted graphene oxide (GO-g-PAA) and then incorporated into PAM networks for GO-g-PAA/PAM preparation. Eventually, the proposed nanocomposite hydrogels were prepared using in situ chemical reduction (Figure 2c). The outstanding characteristics of this study are the enhancement of hydrogen bonds from the interaction between the PAM matrix and grafting of PAA chains, which prompted the reinforcement of interfacial interaction as well as the electrical and tensile properties, swelling behaviors and self-recovery.

#### 2.1.4. Addition of Metal Ions or Hydrophobic Monomer

Hydrophobic monomers or metal ions are also involved in the one-step fabrication of a graphene-incorporated hydrogel, which facilitates the self-assembly of graphene oxide sheets into 3D interconnected networks. Typically, Cong and coworkers alloyed dissolved GO with a certain quantity of FeSO_4_ into a cylindrical vial [77]. Afterward, the pH of the mixture was adjusted and it was laid at 90 °C in an oil bath for 6 h to perform the reaction. Moreover, in another study, they also proposed a novel type of highly elastic hydrogel by blending the PAM network in the prepared GO dispersion solution in the presence of Ca(NO_3_)_2_ as an additive (Figure 2d). In this investigation, Ca^2+^ was coordinated with oxygen-containing groups in the GO sheets, which facilitated GO assembly into a 3D network [78].

Overall, the benefit and drawback of these preparation techniques for graphene integrated hydrogel in term of physical, chemical and biological properties are discussed in Table 1, showing the applicable potentials of different methods for the fabrication of GGels.

### 2.2. Photothermal Property of GGels

**Figure 3:**
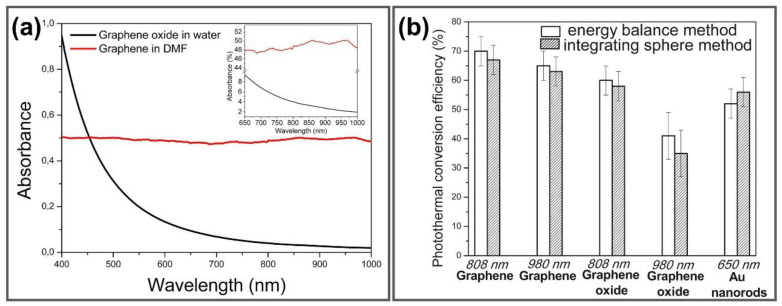
(**a**) Absorption spectra of graphene in dimethylformamide and GO in water in both visible and NIR range, inset is magnification of spectra from 650–1000 nm. (**b**) Photothermal conversion efficiencies of graphene, GO and Au nanorods determined by time constant and integrating sphere methods. Adapted with permission from [80].

Based on an inherently extraordinary structure, graphene can strongly interact with low-frequency photons and generate heat under specific wavelengths such as NIR light through plasmonic photothermal conversion [79]. Upon NIR irradiation, graphene surface plasmons are stimulated and induce random resonance and dipole transmission, which is required for the conversion of thermal photon energy output. The absorption spectra of graphene and GO show that absorbance of GO falls from UV to NIR region while graphene keeps constant absorbance (Figure 3a). To validate the higher thermal conductivity of graphene-based materials for photothermal application, photothermal conversion efficiencies of graphene, GO and Au nanorods were measured under illumination at different NIR wavelength (980, 808, 650 nm) using time constant and integrating sphere methods, respectively, exhibiting the higher photothermal efficiency of graphene and GO about 58–67% under 808 nm NIR irradiation compared to 52% of Au nanorods (Figure 3b) [80]. Hence, GGels also exhibit noticeable photon absorption under NIR irradiation, facilitating local temperature enhancement surrounding injected materials and killing cancerous cells.

Currently, heating is widely used as an effective and useful method for cancer therapy due to the burning of cancerous cells and tumors upon the enhancement of biological molecular temperature. Additionally, for non-superficial treatments, irradiation by laser at NIR was extensively investigated to minimize damage to non-specific surrounding tissues [80]. Among the promising photothermal materials, graphene-incorporated materials have attracted much attention because of their outstanding properties, especially the high absorption of NIR. Therefore, graphene/derivative graphene-based materials have been employed in diverse applications, such as antibacterial and anticancer therapies, drug delivery and tissue engineering. In this context, the photothermal conversion efficiency is the most fundamental factor, which requires only a low material concentration, irradiation power and shorter irradiation time. In particular, in the case of GO, the optical absorption is significantly increased due to stable colloidal suspension formation, eventually enhancing its photothermal conversion efficiency [81]. In comparison with others, for instance, Au nanoshells, nanorods, or Au-Ag alloys, graphene has attracted considerable attention because of its stable shape under high laser power, hence, altering their plasmonic resonances and conserving their photothermal conversion efficiency [82]. Furthermore, hydrogels are considered smart polymers because of their specific stimuli-absorbing capacity, including light and alternating magnetic fields, especially light from NIR laser, to generate and control local heating [83]. Nevertheless, hydrogels can easily alter their reversible structure upon variations in local conditions, such as temperature or pH [84]. To overcome these issues, immobilizing GO nanosheets is the most effective way to not only ameliorate the mechanical properties of the hydrogel, but also accomplish NIR light responsiveness; thus, it has been extensively investigated for biomedical applications [85,86,87]. Benefiting from the excellent photothermal properties of GO and the mechanical properties of hydrogel, GGels also exhibit high photothermal conversion efficiency that converting NIR light into heat and optimal mechanical properties for their promising potential in different photothermal-based biomedical applications.

## 3. Biocompatibility and Bioimaging Properties of GGels

**Figure 4:**
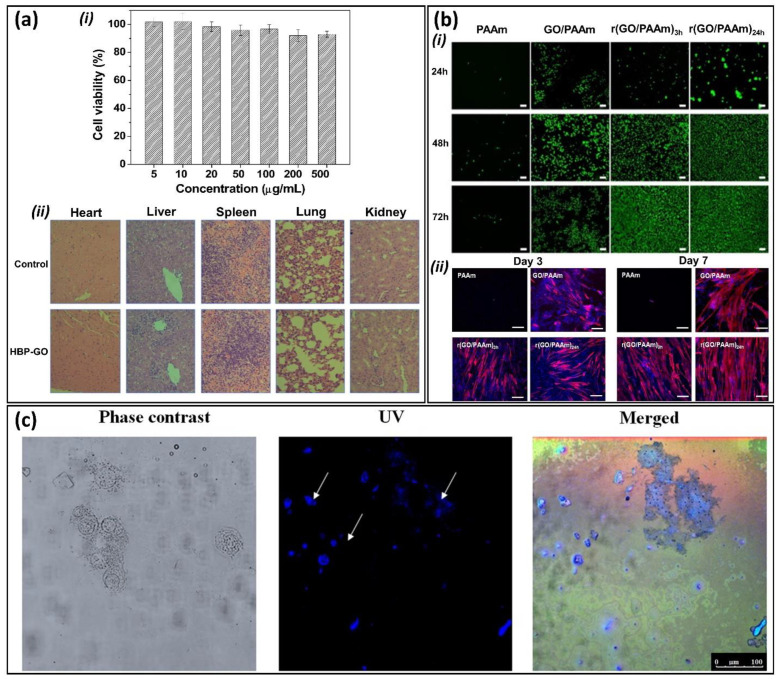
Biocompatibility and bioimaging properties of graphene-based nanocomposites. (**a**) Cytocompatibility of hyperbranched polyglycerol-modified graphene (HPG-GO) on (i) mouse fibroblast NIH-3T3 cell line in vitro at different concentrations of HPG-GO or (ii) in vivo histology of different mouse organs at 500 mg/kg HPG-GO. Adapted with permission from [91]. (**b**) Biocompatibility of different composites of polyacrylamide (PAAm), graphene/PAAm (GO/PAAm) and conductive GO/PAAm hydrogel synthesized after 3 h (r(GO/PAAm)3h) or 24 h (r(GO/PAAm)24h) reduction time on in vitro growth (i) and differentiation (ii) of myoblast C2C12 cells. Adapted with permission from [96]. (**c**) Bioimaging properties of GGels confirmed by incorporation of AgNPs capped GO hydrogels in macrophage using confocal fluorescence microscopy. Adapted with permission from [102].

Graphene-based nanocomposites have been extensively exploited for broad biomedical applications. Nevertheless, according to the existing studies, the biocompatibility and toxicity of graphene remain controversial [88] owing to the complexity and variability in different graphene synthesis procedures, forms or functionalization. The potential biocompatibility and cytotoxicity of graphene are largely associated with the nanomaterial size, shape, morphology, concentration and exposure time [85,89]. The toxicity of graphene to eukaryotic cells has been reported in several studies where cellular membrane integrity was destroyed and excessive generation of reactive oxygen species (ROS) was enhanced [90]. The study by Wang et al. showed that GO at higher than 50 µg/mL caused significant toxicity to human fibroblast cells by entering and disrupting the normal function of the mitochondria, lysosomes, endoplasm and nucleus in vitro and lung granuloma formation in vivo [86]. However, the currently existing data have demonstrated both in vitro and in vivo biocompatibility of graphene and its derivatives [87,91]. In particular, Cheng et al. showed that either β-cyclodextrin aldehyde GO network alone (β-GO) or β-GO-hydrogel does not affect NIH 3T3 cell proliferation and behavior in vitro even after 7 days [92]. Another graphene-based nanocomposite, hyperbranched polyglycerol-modified graphene, was also reported to be highly compatible with either 3T3 cell in vitro at 500 µg/mL of the nanomaterial, or the tissue integrity in vivo, or blood physiology (Figure 4a) [91]. Studies on the mouse model have demonstrated the long-time circulation of GO in the blood and accumulation in the lungs following intravenous administration, leading to induced lung inflammation and granulomatous reaction [85]. Nevertheless, no significant pathological change was observed in the kidney, spleen and liver, which is in accordance with the study by Guangbo et al. [93].

Notably, surface-modified graphene exerts remarkably improved biocompatibility and less toxicity. Surface modification of graphene by poly(acrylic acid) exhibited significantly lower pro-inflammatory and platelet depletion effects and caused no pathological changes in the liver and lungs in the mouse model compared to GO alone [94]. According to this study, the enhanced biocompatibility is associated with the distinct protein corona compositions formed on the surface of functionalized GO, which affects its interaction with the cellular membrane and uptake of GO and the resultant cellular response. More recently, several studies have reported the excellent biocompatibility of graphene-incorporated hydrogels [95,96]. Bacterial nanocellulose/poly(acrylic acid)/GO composite hydrogels were reported to significantly improve fibroblast cell adhesion and proliferation, suggesting their good compatibility and great potential for wound dressing applications [95]. Another conductive GGel fabricated by Park et al. did not cause the recruitment of any inflammatory cells in the in vivo implantation experiment, indicating its tissue compatibility [97]. Currently, conductive hydrogels r(GO/PAAm) generated by a reduction reaction between hydrogels and graphene oxide/polyacrylamide (GO/PAAm) not only exhibited good biocompatibility with the myoblast C2C12 cell line but also supported cell function and differentiation in vitro (Figure 4b) [96]. Graphene was found to significantly enhance the biocompatibility of hydrogel composites [98], suggesting that graphene is not merely a nanomaterial supporting the formation of hydrogel networks, but instead improves the distinct characteristics of hydrogel composites.

Along with the biocompatible properties, the bioimaging properties of GGels owing to graphene fluorescence were evaluated to designate the potential bioimaging applications of these GGels-based biomaterials [99]. GGels exhibit striking fluorescence properties that allow bioimaging; hence, they can make the disease diagnosis and treatment in labeling cells robust [39]. Fluorescent GGels formed by cellulose nanocrystals (CNCs) and graphene quantum dots (GQDs) was developed as injectable material of 3D printing. CNCs-GQDs hydrogels exhibited strong blue fluorescence when excited at 365 nm with weak autofluorescence due to the photoluminescence properties of GQDs [100]. GQDs also acted as crosslinker for carboxymethyl cellulose (CMC) to make biocompatible CMC-GQDs hydrogels that exhibit the fluorescent property for fluorescent bioimaging application. These GQDs showed blue emission at 461.5 nm under the excitation wavelength at 367 nm, making GGels formed from these GQDs emit strong blue fluorescence as a unique photoluminescent property suitable for bioimaging application [101]. The fluorescent bioimaging application of GGels was investigated using capped silver nanoparticles (AgNPs) capped GO nanosheets hydrogels. The cellular incorporation of AgNPs capped GO hydrogels was confirmed by clear blue dots inside macrophage cells using confocal fluorescence microscopy (Figure 4c) [102]. In conclusion, even though more research is required to verify the biocompatibility and bioimaging properties of GGels-based biomaterials prior to their therapeutic application, these nanocomposites could serve as superior materials with excellent mechanical, physical, chemical and biological properties, making them ideal biomaterials for numerous biomedical applications.

## 4. Photothermal-Based Biomedical Applications of GGels

### 4.1. Photothermal Anticancer Therapy

#### 4.1.1. Pure Photothermal Therapy

**Figure 5:**
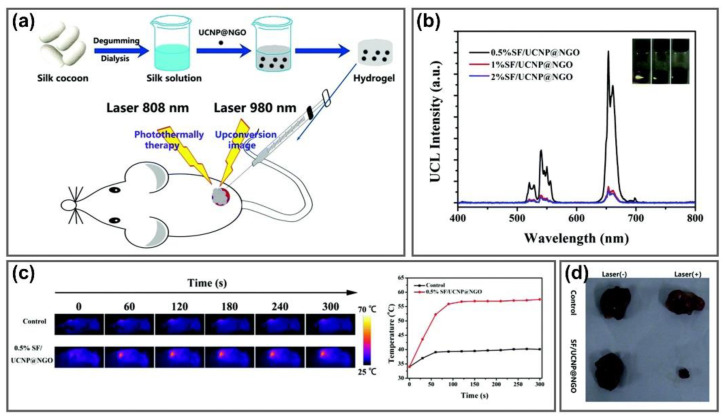
GGels for photothermal anticancer therapy. (**a**) Scheme of synthesis of SF/UCNP@NGO injectable silk fibroin nanofiber hydrogel hybrid system for synergistic upconversion luminescence imaging and PTT of breast cancer. (**b**) Upconversion luminescence spectra and digital pictures (inset) of SF/UCNP@NGO hydrogels at different SF concentrations under 980 nm laser irradiation. (**c**) In vivo photothermal imaging and temperature of tumors injected with PBS and 0.5% SF/UCNP@NGO hydrogels with 808 nm laser irradiation. (**d**) Mice tumors after photothermal treatment with SF/UCNP@NGO hydrogels. Adapted with permission from [110].

Based on its excellent thermal conductivity, GO has been utilized as an effective PTA for formulating GO with hydrophilic polymers to improve the thermal-sensitive properties of hydrogels for PTT of cancer. Upon NIR irradiation, GO can absorb infrared radiation and transfer into thermal energy because of its delocalized electron arrangement, increasing the temperature in cell and tumor tissues, thereby activating the killing activity towards cancer cells and suppressing tumor development [103,104,105]. Graphene/poly (acrylic acid) (PAA)/clay nanocomposite hydrogels were fabricated via an in situ polymerization approach using acrylic acid as the monomer and graphene and clay nanosheets as the additive. Graphene was utilized as an effective additive to enhance the self-healing ability and photothermal properties of PAA/clay hydrogels. The graphene/PAA/clay hydrogel strongly improved the self-healing ability of hydrogels compared to the hydrogels without graphene, which superfast healed within 30 s by adding only 0.16 wt% graphene. Upon 808 nm NIR exposure, the central temperature of the graphene hydrogel increased rapidly to 28.7 °C within 1 s and reached 50.2 °C after 30 s compared to that of the PAA/clay hydrogel, indicating the strong photothermal conversion efficiency and thermal conductivity of hydrogels from graphene [106]. Lee et al. illustrated wavelength-independent photothermal hydrogelation using PEGylated GO (GO-PEG), which showed good heat generation and excellent colloidal stability under a wide range of wavelengths. NIR light irradiation at 532, 785 and 980 nm increased the temperature of the GO-PEG solution to 43 °C and subsequently initiated the thermal radical polymerization of PEG diacrylate (PEGDA). The hydrogels formed by GO-PEG exhibited similar mechanical properties with different lasers (532, 785 and 980 nm) compared to specific 532 nm AuNPs and 785 nm AuR-based photothermal gelation systems. The GO-PEG-based photothermal hydrogelation of PEGDA was successfully employed for remote transdermal gel formation in vivo with 785 and 980 nm lasers, suggesting a promising approach for the biomedical application of light-inducible and injectable hydrogels [107]. Upconversion nanoparticles (UCNPs) upconverting NIR laser into UV light are widely exploited as luminescence probes for in vivo imaging because of their unique properties including deep tissue penetration, low absorption and scattering of bio-tissues and organs, good photostability and non-photoblinking or -photobleaching [108,109]. To achieve the combined effect of upconversion luminescence imaging diagnosis and PTT, He et al. developed a silk fibroin nanofiber (SF) hydrogel system by doping the NaLuF_4_:Er^3+^,Yb^3+^UCNPs and nano-graphene oxide (nGO) into SF solution to form SF/UCNP@nGO. This hydrogel exhibited negligible toxicity to cancer cells under laser irradiation, which could be used in PTT (Figure 5a). The strong luminescence of UCNPs at 520, 540 and 653 nm emission wavelength upon 980 nm excitation was utilized as an imaging agent for in vivo imaging with intratumor injection in a white mouse (Figure 5b), while the temperature of the SF/UCNP@nGO hydrogels increased to 60°C after irradiation with 808 nm NIR laser due to the photothermal property of nGO that was used for in vivo PTT (Figure 5c). With the treatment with 0.5% SF/UCNP@nGO hydrogels on the 4T1 tumors of mice, the tumor temperature increased to 57°C after 120 s of irradiation with an 808 nm laser, which is sufficient to induce the death of cancer cells and completely suppress tumor growth after the therapeutic period (Figure 5d) [110]. These results suggest that GGels could be used as an ideal material for thermotherapy applications.

#### 4.1.2. Thermal-Triggered Drug Delivery

**Figure 6:**
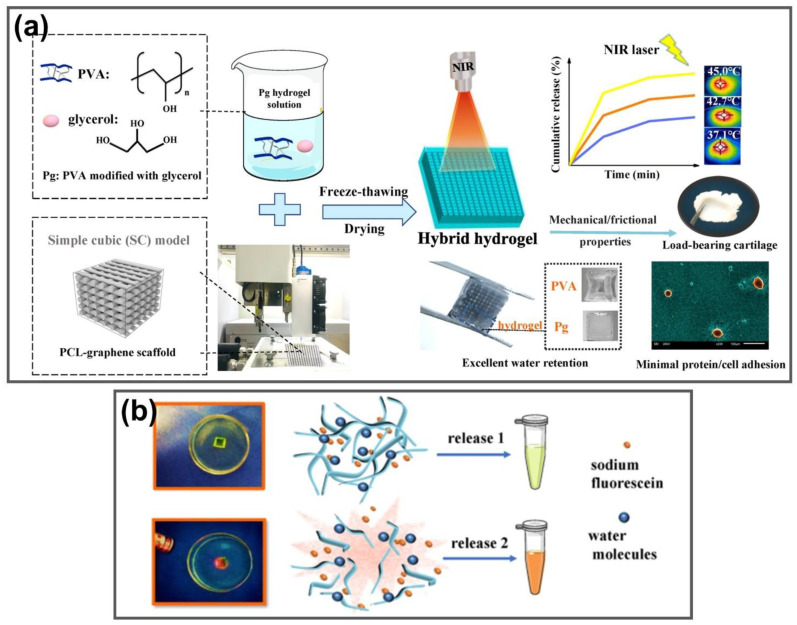
GGels for NIR-responsive drug delivery systems in anticancer treatment. (**a**) Schematic illustration of the fabrication and characteristics of PG-Pg hybrid hydrogels. (**b**) Drug release mechanism of PG-Pg hydrogels without and with NIR irradiation. Adapted with permission from [116].

Graphene and its derivatives possess unique physicochemical properties including acceptable biocompatibility, cargo loading capacity, ultrahigh surface area, straightforward bio-functionalization and considerable potential for drug delivery applications [111,112,113,114,115]. GGels have been explored as PTAs in NIR-triggered drug delivery systems [116,117,118]. Jiang et al. developed a load-bearing system consisting of a glycerol-modified polyvinyl alcohol (PVA) hydrogel reinforced by a 3D printed PCL-graphene scaffold named PG-Pg. This hydrogel formed by reinforcement with a 3D printed PCL-graphene scaffold with optimized architecture demonstrated desirable mechanical properties (stiffness, toughness and tribological properties) matching those of natural load-bearing cartilage. The PG-Pg hydrogel was designed to provide drug release and on-demand photothermal conversion functions with excellent biocompatibility and low cell adhesion (Figure 6a). The graphene-containing 3D printed reinforcing scaffold provides a hybrid system with effective photothermal conversion properties, which can regulate drug release under external laser irradiation. Under NIR exposure, PG-Pg hydrogels generated heat to induce greater molecular mobility and then promoted drug release by accelerating the transport of both water and drug molecules (Figure 6b). The hybrid hydrogel system also allows for minimal protein adsorption and cell adhesion, further demonstrating its excellent potential for load-bearing cartilage applications [116]. Wang et al. fabricated chitosan-modified chemically reduced GO (CrGO) incorporated into a nanogel (CGN) as a thermosensitive nanogel for NIR-triggered drug delivery applications. This nanogel displayed reversible NIR-induced thermal effects from 25–42 °C, as well as high doxorubicin (DOX) loading capacity (48 wt%). Upon stimulation with 808 nm NIR laser, the nanogel temperature reached 42 °C, leading to a DOX released capacity to increase to 18.81% after five cycles of NIR exposure to DOX (3 min interval), which was significantly higher than that of approximately 5% without irradiation due to the thermo-sensitive release of DOX. The Lewis lung cancer 1 (LLC1) cell line and mouse prostate cancer cell line (TRAMP-C1) exhibited remarkably greater cytotoxicity with the combination of photodynamic and photothermal effects from DOX and rGO, with only 51.31% cell survival, compared to 95% cell viability without NIR irradiation. These results demonstrate the promising application of CGN for on-demand drug release by NIR light. Upon exposure to the NIR laser, the rGO was absorbed and converted to heat, increasing the nanogel temperature, consequently leading to shrinkage and release of the drug, indicating that these GO-based nanogels could act as effective photo-chemo therapeutic agents for NIR-triggered on-demand drug release [117]. An 87 nm thermo/pH/magnetic-triple sensitive nanogel consisting of magnetic GO with excellent temperature, pH and the magnetic responsive ability for anticancer drug delivery. Upon the treatment with cancer environment pH, temperature and magnet, DOX anticancer drugs loaded into nanogels increased the release rates due to the pH decrease, temperature increase and presence of the magnet, resulting in a decrease in viability of Michigan Cancer Foundation-7 (MCF-7) cancer cells [118].

#### 4.1.3. Synergistic Photothermal Cancer Therapy

**Figure 7:**
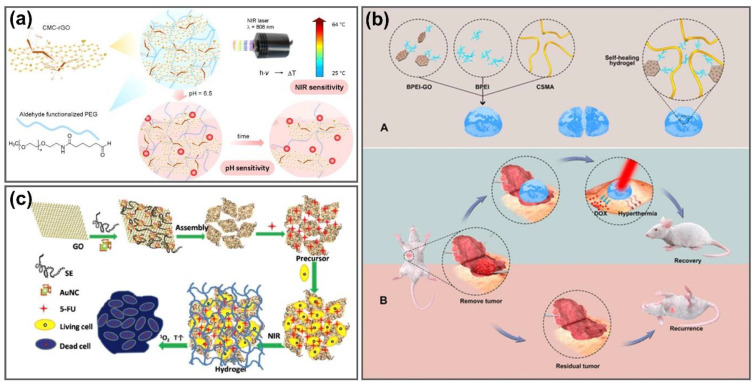
GGels for synergistic PTT. (**a**) Fabrication of carboxymethyl chitosan-functionalized GO hydrogels for chemo-photothermal synergistic therapy system with efficient drug release upon dual stimulation of NIR light and pH. Adapted with permission from [39]. (**b**) Development of CSMA/BPEI/BPEI-GO hydrogels for synergistic chemo-PTT to prevent locoregional recurrence of breast cancer. Adapted with permission from Ref. [129]. (**c**) Formation process of rGO/AuNCs/SE hydrogel synthesis, 5-FU drug loading and combined photothermal/photodynamic/chemo synergetic antitumor. Adapted with permission from [137].

Reasonably, the combination of PTT and chemo- or/and photodynamic therapy (PDT) could enhance the synergistic therapeutic efficiency against cancer. Different from PTT that kills the tumor based on the heating effect after NIR absorption, PDT destroys cancer cells by reactive oxygen species generated from photochemical reactions due to utilization of non-toxic photosensitizers that are activated to form the excited singlet state and transformed to excited triplet state upon irradiation of visible light [119]. Hence, synergistic anticancer therapies have attracted the attention of researchers to develop highly effective treatments by taking advantage of the photothermal conversion property of graphene, chemotherapeutic drug-based hydrogels and photodynamic nanomaterials [120,121,122,123]. Liu et al. developed an NIR- and pH-responsive carboxymethyl chitosan-functionalized reduced GO/aldehyde-functionalized poly (ethylene glycol) (CMC-rGO/CHO-PEG) hydrogel that exhibits a highly efficient delivery performance of DOX antitumor drugs for combined chemo-PTT in mouse connective tissue fibroblasts (L-929) (Figure 7a). To obtain a good distribution and strong NIR absorption property, GO nanosheets were functionalized with CMC to form the CMC-rGO complex, leading to enhanced photothermal performance of the CMC-rGO/CHO-PEG hydrogel by three times compared to that of the CMC/CHO-PEG hydrogel. The temperature of the as-prepared hydrogel reached above 50 °C within 8 min after NIR irradiation, significantly higher than that of the CMC/CHO-PEG hydrogel under the same NIR irradiation condition. DOX was also loaded into the hydrogels to provide a pH-sensitive release of anticancer drugs for chemotherapy. DOX release from the hydrogel approached 99% in the cancerous biological environment, which was much more efficient than that in a physiological environment. There was a negligible effect on drug release either at pH 7.4 or 6.5 under 808 nm NIR irradiation, indicating that NIR irradiation could not improve the drug release capacity due to the strong interactions of DOX with rGO in the CMC-rGO/CHO-PEG hydrogel [39]. Although NIR exposure could not improve the drug release capacity, this hydrogel with chemo-photothermal efficiency was successfully used in cancer chemo-PTT applications. To enhance the drug loading and release efficiency, Xu et al. also fabricated nanogels by incorporating GO nanoplatelets into an alginate polymer using a disulfide molecule as a crosslinker, which exhibited a high encapsulation efficiency of DOX anticancer drugs via electrostatic interactions with 97.2% wt. These nanogels were then applied to stimulate the death of A549 cells (adenocarcinoma human alveolar basal epithelial cell line) due to the dual chemotherapeutic and photothermal therapeutic effects against cancer cells with 808 nm NIR laser. The anticancer bioactivity of these nanogels was enhanced by increasing the temperature from 25–50 °C after 4 min 808 nm NIR laser exposure to suppress the cells [124]. The hyaluronic acid/polyaspartamide-based nanogels were reported by Fiorica’s group for the treatment of colorectal carcinoma [125]. The double-network-structured nanogel was fabricated by the addition of an amino-functionalized hyaluronic acid (HA) with an acryloyl-functionalized polyamino acid in the presence of single-layer GO flakes. Owing to the large aromatic surface area of GO, a water-soluble antitumor drug, irinotecan, can be easily loaded onto nanogels with high amounts (33.0% w/w). HA, which is recognized by CD44 and RHAMM glycoproteins, overexpressed receptors of colon cancer cells, were utilized as colloidal carriers for target-specific delivery of anticancer drugs [126,127]. Hundred percent hyperthermia-mediated irinotecan release occurred after 4 h of incubation with 810 nm NIR laser irradiation. Through the synergistic hyperthermic/cytotoxic effect, this nanogel was taken up by human colon cancer cells (HCT 116), which then produced the photothermal therapy to accelerate the death of colon cancer cells [125]. Another functionalized thermosensitive GGel was developed by Zhu et al. to enhance therapeutic efficacy and alleviate system toxicity. The stability of GO in physiological solutions was improved by functionalization of GO with chitosan (CS). Docetaxel, a member of the taxane family of antineoplastic agents for anticancer activity, was loaded into the GO/CS dispersion, followed by mixing a docetaxel-graphene oxide/chitosan suspension with a hydrogel made from Poloxamer 407 and Poloxamer 188 to fabricate the hydrogel. The biocompatible Poloxamer 407 and Poloxamer 188 have photothermal sensitivity that can be used to control drug release capacity [128]. Docetaxel–graphene oxide/chitosan hydrogels exhibited higher antitumor efficacy in MCF-7 cells in vitro and tumor tissues of mice in vivo with minimal side effects without obvious toxic effects, significantly improving the cancer chemo-PTT through 808 nm NIR laser irradiation [121]. Graphene nanoparticle-based self-healing hydrogels were synthesized via a Schiff-base linkage consisting of chondroitin sulfate multialdehyde (CSMA), branched polyethylenimine (BPEI) and BPEI-conjugated graphene (BPEI-GO) to prevent postoperative recurrence of breast cancer (Figure 7b). BPEI stabilized the structure while GO provided sustained drug delivery and NIR-triggered photothermal effects, exhibiting excellent self-healing (∼100%) and improved mechanical properties (7000 Pa). The photosensitive properties of these hydrogels were confirmed by the significant temperature increase in CSMA/BPEI/BPEI-GO compared to CSMA/BPEI. The hydrogels were loaded with DOX and injected into breast cancer MCF-7 cells (human breast adenocarcinoma cell line) in vitro and in a cancer mouse model in vivo to investigate the cell killing efficiency via synergistic chemo-PTT. To prevent postoperative recurrence in a breast cancer mouse model, the chemo-photothermal therapeutic effects of CSMA/BPEI/BPEI-GO hydrogels with NIR laser irradiation reduced tumor recurrence to 33.3%, compared with 66.7% without NIR treatment, suggesting that the exploitation of CSMA/BPEI/BPEI-GO hydrogels can achieve high anticancer activity to prevent postoperative recurrence in breast cancer [129]. In situ injectable chitosan-agarose that form thermo-responsive hydrogels through the incorporation of GO (thermogel-GO) or rGO (thermogel-rGO) that showed suitable injectability and gelation time, as well as good physicochemical properties and cytocompatibility, were prepared. Upon 808 nm NIR irradiation, the temperature of thermogel-rGO increased by 3.8-fold compared to the thermogel-GO because of the photothermal improvement in rGO, enhancing the PTT effect up to 60% cell viability, while that of the thermogel-GO only reduced to 73% after laser treatment. With the combination of DOX and ibuprofen (IBU) with thermogel-rGO, the chemo-photothermal anticancer effect was enhanced by diminishing the MCF-7 cell viability to 34%, making thermogel-rGO effective for chemo-PTT of cancer cells [40]. Photothermal hydrogelation using NIR light and PTAs with good tissue penetration has been exploited to improve the injectable hydrogel system by providing in vivo controllability of the gelation process [130,131,132]. Plasmonic nanoparticles were utilized as photothermal agents; however, the specified photothermal heating ability of plasmonic nanoparticles within their localized surface plasmon resonance peaks limits their photothermal gelation with plasmonic nanoparticles as a heating material [133,134]. Sahu et al. reported a self-assembled hydrogel formed from GO nanosheets and pluronic without the chemical modification of GO. Pluronic chains act as physical cross-linkers among GO sheets because of their adsorption onto GO nanosheets via hydrophobic association and interaction with nearby GO sheets via H-bonding, contributing to the strength of the hydrogel by different concentrations and types of pluronic. GO nanosheets that absorb near-IR laser at 808 nm can trigger rapid gel formation within 30 s by the photothermal effect. The synergistic effect of the heat capacity of GO and temperature-induced hydrophobicity increase of pluronic helps the thermosensitive hydrogels form a solid state. This hydrogel shows stable gel formation after mice injection without a chronic inflammatory response, making it applicable as an injectable gel system for biomedical applications [135]. A pluronic F127 (F127)-stabilized GO supramolecular hydrogel was developed as a safe nanovehicle for chemo-PTT. DOX can be loaded non-covalently on the F127-GO hydrogel by strong π–π interactions, hydrophobic interactions and the strongest hydrogen bonding. The supramolecular hybrid hydrogel exhibited an excellent drug-loading content of DOX (100%) and improved drug release compared to F127-DOX. The A549 cell inhibition rate of F127-GO-DOX after 808 nm laser exposure was increased to 60%, which was higher than the 40% inhibition rate of non-drug-loaded carriers due to the synergistic effects of chemo-PTT. In an in vivo study, F127-GO-DOX effectively suppressed tumor growth under NIR treatment after 14 days, indicating that the strong photothermal effect of GO significantly increased the chemo-PTT therapeutic effect of this hydrogel under NIR laser [136].

Gold nanomaterials have been utilized to improve the thermal-responsive therapeutic efficiency of GGels as PTT mediators owing to their properties, including strong photothermal conversion, high biocompatibility and straightforward surface modification [138,139,140]. Chang’s group utilized amaranth extract (AE) as both a reductant and cross-linking agent to fabricate a composite rGO/AE/AuNP hydrogel in a PDT/PTT-integrated platform for enhanced cancer therapy. The chlorophyll derivatives in AE act as PDT drugs, while the combination of rGO and AuNPs exhibit stronger photothermal effects and accelerates the generation of cytotoxic singlet oxygen (^1^O_2_). The rGO/AE/AuNPs hydrogel significantly increased the temperature by 6.3 °C under 808 nm NIR irradiation at 200 mW·cm^−2^ power density, compared to only 0.9 °C temperature increase of water under the same condition. The formation and regulation of the hydrogel shell on HeLa cells or Chinese hamster ovary cells easily occurred under NIR irradiation within 10 min and did not only leave a high concentration of drugs at the lesion site, but prevented them from migrating to normal tissues that could reduce the side effects. This hydrogel exhibited remarkable PDT/PTT effects for synergistic therapeutic effects in anticancer therapy [122]. Another composite hydrogel containing spinach extract (SE), rGO and gold nanocages (AuNCs) was prepared for the combination of localized drug release with multimodal therapy of malignant tumor (Figure 7c). SE did not only form hydrogel but also played a role in the improvement of hydrogel biocompatibility as well as killing tumor cells as a natural photosensitizer under 660 nm laser radiation. AuNCs exhibit obvious photothermal properties and enhance ^1^O_2_ generation. Upon loading with fluorouracil (5-FU), good drug retention ability and controllable release of 5-FU were observed in a neutral physiological environment and the acidic environment of tumor cells, respectively. After 10 min of NIR irradiation, the survival rate of HeLa cells incubated with 5-FU loaded hydrogel sharply decreased to 1.2% due to the synergistic photothermal/photodynamics/chemo therapeutic effect, making it an excellent delivery carrier for combined PTT/PDT/Chemo cancer treatment [137]. A topical hydrogel patch containing a polymer hydrogel and hybrid nanostructures was fabricated by Matai’s group for nanomaterial-assisted photothermal therapeutics and drug delivery applications. Gold nanorods (AuNRs) were decorated onto polyvinylpyrrolidone (PVP)-functionalized GO (PVP-nGO) to form PVP-nGO@AuNRs hybrids via electrostatic interaction between the opposite charges of PVP-nGO and CTAB-AuNRs, followed by dropwise addition of these hybrid nanostructures to alginate (Alg) and polyacrylamide (PAAm) mixture to successfully synthesize PVP-nGO@AuNRs/Alg/PAAm patch with a pore size of ~28.60 ± 3.10 μm. The photothermal properties of the hybrid patch were characterized under 888 nm NIR exposure that increased the surface temperature to 59.7 °C and 75.16 °C within 5 and 10 min, respectively, which are adequate for thermal suppression of cancer cells. The heat generated from the hybrid patch could be transmitted to mimic skin tissue without much loss while retaining the photothermal stability for multiple cycles. Within 20 min of stimulated NIR irradiation, the drug-loading hybrid patch exhibited a higher capacity of drug release of up to 30% of methotrexate (MTX, water-insoluble anticancer drug) and 43% of rhodamine B (RhB, a water-soluble anticancer agent), compared to 9% of MTX and 20% of RhB without laser exposure. This nanomaterial-loaded hydrogel patch exhibits promising potential for the development of externally placed hydrogel patches for thermal destruction of localized solid tumors and tunable delivery of chemotherapeutic drugs at the target site, suggesting strong implications for mediating targeting, shrinkage and prevention of tumor recurrence in localized solid tumors [123].

### 4.2. Photo-Inspired Antimicrobial and Wound Healing Acceleration

**Figure 8:**
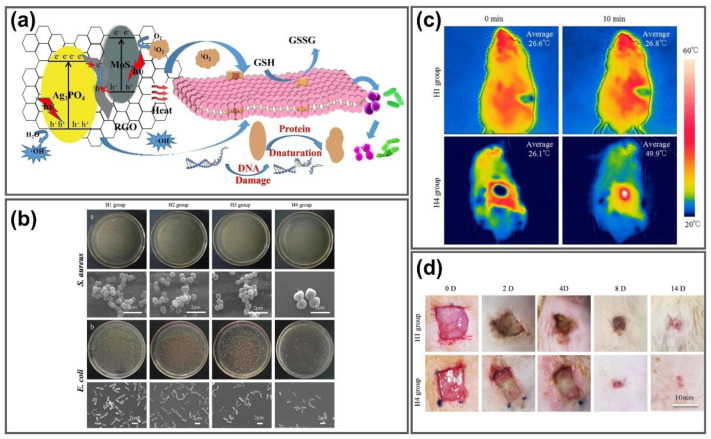
GGels for antibacterial and wound healing application. (**a**) ROS generation mechanism of rGO/MoS_2_/Ag_3_PO_4_ composite hydrogels and inactivation mechanism for bacteria under light irradiation. (**b**) Representative colony-forming unit images and SEM images of *S. aureus* and *E. coli* of in vitro antibacterial assay of different hydrogels (H1 group: pure hydrogel; H2 group: GO hydrogel; H3 group: rGO/MoS2 hydrogel; H4 group: rGO/MoS_2_/Ag_3_PO_4_ hydrogel). (**c**) In vivo thermal imaging of hybrid hydrogels on the back of the rats under 0 min and 10 min exposure of dual lights. (**d**) In vivo wound healing effects of composite hydrogels in the rats after different treatment durations. Adapted with permission from [142].

Bacteria-infected wounds are attracting increasing attention, as antibiotic misuse and multidrug-resistant bacteria complicate their treatment. Hydrogel-based dressings can provide a moist environment for wounds, exhibiting beneficial advances for wound healing. GGels have been exploited as effective antibacterial materials that can be further utilized in wound healing applications [141,142,143]. Zhao et al. fabricated 3D hemin-functionalized graphene (Hem/GO) hydrogels via π–π interactions using a self-assembly approach for strong antibacterial applications. The Hem/GO hydrogels not only exhibited good mechanical strength (609–642 kPa storage modulus) and high adsorption capacity for RhB (341 mg/g) owing to their large volume and high porosity, but also enhanced photocatalytic degradation efficiency towards methylene blue (MB) through the participation of O_2_^•−^ radicals even after excellent cycling performance, making it a strong bioactive agent for photodynamic inactivation of microorganisms. Upon MB photodegradation, visible-light irradiation induced a pH solution change from alkaline pH 8.99 to acidic pH 3.82 to accelerate the generation of acidic degradation products, providing strong antibacterial properties to *Bacillus subtilis* with 46% surviving fraction and is completely invisible after 30 min and 150 min light irradiation [144]. Zhang et al. synthesized a PVA hydrogel incorporated with rGO/MoS_2_/Ag_3_PO_4_ composites for highly efficient sterilization during wound healing under light irradiation (Figure 8a). The rGO/MoS_2_/Ag_3_PO_4_ composite was prepared by a two-step process containing hydrazine reduction and in situ deposition, then doped into hydrogels to enhance the mechanical properties and swelling ratio. The hybrid hydrogel exhibited excellent antibacterial properties against *Staphylococcus aureus* and *Escherichia coli* under co-irradiation with 660 nm visible light and 808 nm NIR light with 97.8% and 98.33% antibacterial rates within 10 min, respectively, due to the synergistic photodynamic and photothermal effects from the rGO/MoS_2_/Ag_3_PO_4_ hydrogel, which were higher than those of rGO and rGO/MoS_2_ hydrogels, which was confirmed through SEM morphologies of *S. aureus* and *E. coli* in Figure 8b. The in vitro thermal images of rats treated with composite hydrogels and pure hydrogels indicate a significant increase in the temperature of the rGO/MoS_2_/Ag_3_PO_4_ hydrogels after 10 min 808 nm laser irradiation (Figure 8c). Compared to the pure hydrogel, the rGO/MoS_2_/Ag_3_PO_4_ hydrogel displayed better healing effects and effectively reduced bacterial infection-related inflammation during wound healing (Figure 8d), indicating that this rGO/MoS_2_/Ag_3_PO_4_-incorporated hydrogel is a promising material for antibacterial wound healing dressing [142].

**Figure 9:**
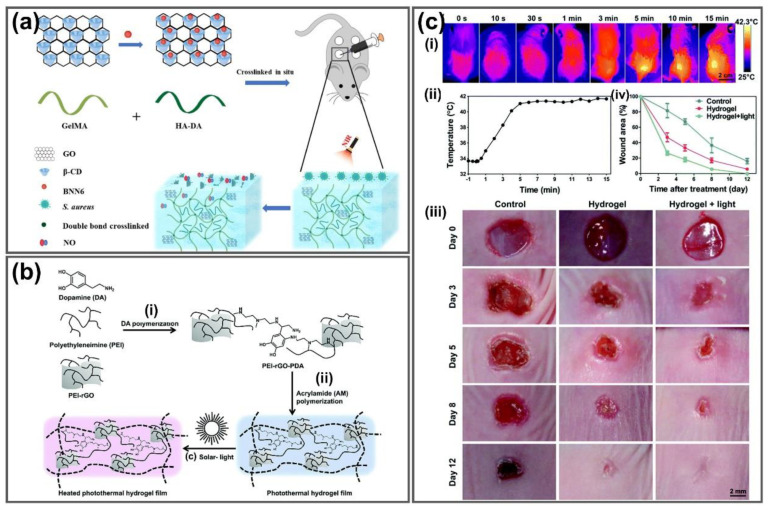
(**a**) Illustration of fabrication of GO-βCD-BNN6 hydrogels as an NIR-mediated NO release nanovehicle for bacteria-infected wound healing. Adapted with permission from [143]. (**b**) Preparation process of photothermal hydrogel film from (i) the polymerization of DA in the presence of PEI and PEI-rGO to (ii) polymerization of acrylamide and heat generation by this hydrogel film under solar light exposure. (**c**) In vivo wound healing effects of photothermal hydrogel film on mice after 12 days of treatment. (i) thermal images of GGel-treated mice under solar light, (ii) photothermal heating curve of wound site during 15 min exposure, (iii) skin wound photographs after 12 days treatment, (iv) wound area measurements of mice after treatment of different groups. Adapted with permission from [145].

Huang et al. developed a methacrylate-modified gelatin (GelMA)/HA-DA hydrogel consisting of β-cyclodextrin (βCD)-functionalized GO and BNN6 (Figure 9a). BNN6 is a Bis-N-nitroso compound as a high-thermal-stable nitric oxide (NO) donor that can be triggered by NIR to produce more NO, generating hydrophobic radicals that damage DNA for antibacterial applications. The GelMA/HA-DA/GO-βCD-BNN6 exhibited good swelling ability (417%), stable photothermal effects (about 60 °C after several cycles of irradiation), NO release of up to 4 µM NO and antibacterial activity against *S. aureus* and *E. coli* bacteria (97.6 and 95.5% inactivation rate) under 808 nm NIR irradiation. Additionally, collagen deposition, angiogenesis and wound healing were promoted by these hydrogels in a mouse model of full-thickness skin repair, compared to the commercial Aquacel Ag dressing, making it a potential material to enhance the regeneration of bacteria-infected wounds [143]. Liang’s group utilized HA-graft-dopamine (HA-DA) and rGO to fabricate an adhesive photothermal antibacterial hydrogel using H_2_O_2_/HPR (horseradish peroxidase) as an initiator system for wound healing and skin regeneration. HA has good biocompatibility, biodegradability, moisture retention and gelation properties, which favor the design of injectable hydrogel dressings, while polydopamine (PDA) possesses good wet adhesiveness to soft tissues. Grafting dopamine (DA) to HA supplied good tissue adhesion and good antioxidant ability, improving the hemostatic effect for multifunctional injectable hydrogel dressing. The obtained rGO not only enhanced the dispersity and electrical conductivity, but also possessed excellent photothermal antibacterial properties that contribute to the effect of wound dressing. These hydrogels exhibit a high swelling capacity of up to 305.5%, degradability with a fast ratio of 50.2% and superior mechanical properties to human skin with good stretchability (93.9–200.8%), tensile stress (65.1 kPa) and good fatigue resistance. Compared to the commercial Tegaderm film and HA-DA/rGO hydrogel without rGO/PDA, the rGO@PDA added hydrogel exhibited a more efficient in vivo wound healing effect in terms of wound closure, granulation thickness and collagen metabolism. Hydrogel dressings not only significantly enhance vascularization by upregulating growth factor expression of CD31 but also improves granulation tissue thickness and collagen deposition, promoting wound closure and better therapeutic effect than Tegaderm films. With sustained drug release, these adhesive hemostatic antioxidative hydrogels could complete skin regeneration, making them excellent wound dressing for full-thickness skin repair [141]. A photothermal hydrogel film with self-adhesive properties was developed by the photo-initiated polymerization method by taking advantage of the excellent adhesion of bioinspired PDA and the great photothermal conversion capability of rGO upon solar light irradiation for accelerating wound healing under solar light irradiation (Figure 9b). The formed photothermal hydrogel film could adhere to wounds and convert solar light into heat, warming up the wound locally. The hydrogel film exhibited strong antibacterial properties against *E. coli* and *S. aureus*, with antibacterial activity of 53.8% and 85.7%, respectively. Under solar light irradiation, the localized temperature enhancement (up to 42 °C after 5 min) significantly promoted wound healing with complete wound closure, compared to 5.7% and 16.1% unclosed wounds after 12 days by reducing the inflammatory response and enhancing re-epithelialization, angiogenesis and collagen deposition, presumably correlated with the increased expression of transforming growth factor-β1 (TGF-β1) and vascular endothelial growth factor-A (VEGF-A) (Figure 9c). This photothermal hydrogel film exhibits great potential for accelerating wound healing with the assistance of solar light [145]. 

### 4.3. Tissue Repair and Bone Regeneration

**Figure 10:**
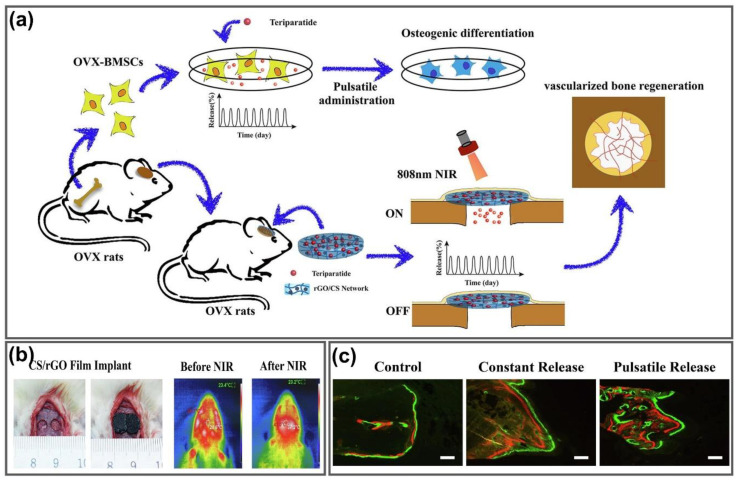
GGels for tissue repair and bone regeneration. (**a**) Schematic diagram of photothermally triggered biomimetic drug delivery of teriparatide via rGO loaded CS hydrogel for osteoporotic bone regeneration. (**b**) Implant of CS/rGO hydrogel films into calvarial defect models of osteoporotic rats and photothermal images before and after low-power NIR irradiation. (**c**) Bone formation after 6 weeks treatment confirmed by the sequential fluorescent labeling of alizarin red (red) and calcein (green). Adapted with permission from [149].

Graphene scaffolds as innovative biomaterials with regenerative properties have been created for tissue engineering and bone regeneration owing to the unique properties of graphene including high growth factor loading, induction of cell differentiation, NIR meditated PTT and the mimic capacity of the strength, stiffness and mechanical behavior of natural bone [146,147,148]. Wang’s group proposed the concept of teriparatide time and dose manner (a drug for osteoporosis treatment) on bone marrow mesenchymal stem cells derived from ovariectomized rats (OVX-BMSCs) for bone regeneration through an osteogenic and angiogenic activity using GO loaded CS hydrogel (CS/rGO) films (Figure 10a). CS/rGO films were fabricated through electrodeposition, then loaded with teriparatide for local drug delivery that mimics pulsatile secretion in physiological conditions due to the photothermal conversion effect. To confirm the effects of these hydrogels on in vivo osteogenesis and angiogenesis, calvarial defect models of osteoporotic rats were implanted with CS/rGO hydrogel films and exposed to NIR laser to achieve the photothermal conversion effect (Figure 10b). Bone generation effects were confirmed through the evaluation of new bone formation based on fluorescent labeling. The new bone area labeling with alizarin (15.18 ± 1.10%) and calcein (19.68 ± 3.38%) was significantly greater than the control group (3.15 ± 0.71%) and constant release group (6.99 ± 0.79%), as shown in Figure 10c. NIR light-responsive CS/rGO films exhibited remarkable effect for bone regeneration through biomimetic delivery of teriparatide and created higher density of blood vessels between the newly formed bone and in the center of defect region, providing the improvement of osteoporotic bone regeneration with minimized unintended systemic side effects [149]. A self-assembled nano-hydroxyapatite (nHA)- rGO scaffold was fabricated with 20 wt% nHA-rGO sheet to form the most stable hydrogel for effective treatment of large tumor-related bone defects. Upon 20 min exposure of 808 nm NIR laser, the nHA-rGO scaffolds killed all but 8% of osteosarcoma cells (MG-63) in vitro; thus, xenografted tumors decreased in vivo after PTT treatment because the NIR laser triggered an increase in temperature of tumors implanted with nHA-rGO scaffolds up to 60 °C after 4 min of irradiation. In addition, the proliferation and osteogenic mineralization of rat bone marrow stem cells (rBMSCs) were promoted by nHA-rGO scaffolds, leading to bone regeneration in rat cranial defects. After 8 weeks, 35% of the cranial defect area remained and bone mineral density reached 284.58 ± 20.78 mg/cm^3^ using nHA-rGO scaffolds, while 80% remained and only 96.04 ± 2.67 mg/cm^3^ was for the control, suggesting that nHA-rGO scaffolds provide effective treatment of large tumor-related bone defects based on their excellent photothermal and osteogenic effects [150]. Lu et al. fabricated the self-supporting graphene hydrogel (SGH) film that enhanced cell adhesion, spreading and proliferation and exhibited therapeutic effects in bone regenerative medicine. The graphene hydrogel film could induce the regeneration of bone without toxic side effects and any additional inducer requirement. The advanced properties of SGH film consisting of osteoinductivity, appropriate mechanical strength and degradability make it potentially attractive for biomedical applications, especially as a “smart” biomaterial for bone regeneration [151]. Therefore, GGels hold a promising potential to become the next generation scaffold for tissue engineering and bone regeneration.

### 4.4. Other Biomedical Applications

**Figure 11:**
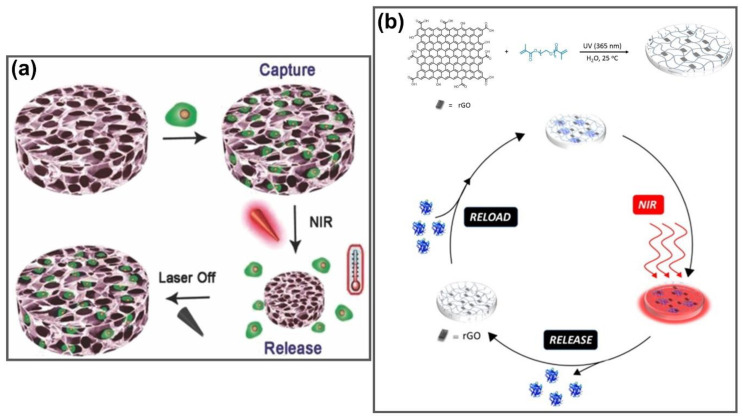
(**a**) Reversible loading and on-demand release of cells using 3D macroporous pNIPAAm-co-pAAm/GO vehicle under on/off NIR laser. Adapted with permission from [153]. (**b**) Fabrication of PEGDMA-rGO hydrogels with efficient loading and photothermal release of insulin for the treatment of diabetes mellitus. Adapted with permission from Ref. [154].

Owing to their advanced properties, GGels have been developed for use in many other biomedical applications such as supporting biomaterials for cell adhesion and growth [152], cell scaffolds for cell preservation, therapeutic cell delivery and disease-related cell isolation and analysis [153], insulin loading and release in the treatment of diabetes [154]. 3D hybrid aerogels formed from GO and black phosphorus nanoflakes (BPNFs) were fabricated for biomedical applications. They exhibited enhanced photothermal properties and electrical properties due to the incorporation of BP compared to that of the neat GO aerogel, which possessed exceptional photothermal stability under ambient conditions without any protection, paving the way to develop GO/BPNF aerogels for promising bio-related applications [155]. In contrast, 3D GO hydrogels were successfully fabricated as an active cell scaffold for reversible cell capture and on-demand release (Figure 11a). GO acted as an NIR-responsive smart element for dynamic control of cell release by changing the morphology and hydrogel function via NIR trigger. Heat generation occurred because of the photothermal effect of GO under NIR exposure, accelerating the transition of hydrogels from hydrophilic swollen to hydrophobic collapsed state, finally promoting the release of cells from the hydrogel. When the NIR light was switched off, the deformed hydrogel returned to its original swollen shape, which could be used for additional cycles. GO hydrogels can efficiently capture cells not only through the bioadhesive ligand arginine–glycine–aspartic acid oligopeptide, but can also release the cells on-demand on the application of an NIR light stimulus, which can realize better dynamic control on cells than traditional passive cell depots, making it a potential NIR-triggered reversible platform for cell preservation, therapeutic cell delivery and disease-related cell isolation and analysis [153]. Furthermore, the formulation of rGO-impregnated poly(ethylene glycol) dimethacrylate-based hydrogels (PEGDMA-rGO) was described for the efficient loading of insulin in photothermally triggered release in diabetic patients (Figure 11b). PEGDMA-rGO hydrogels displayed highly efficient insulin release of approximately 80% by 980 nm NIR-induced heating irradiation, which has no effect on the biological and metabolic activities of the released insulin, providing another opportunity for the treatment of diabetes mellitus [154]. Owing to their excellent physical and chemical properties, GGels have been expanding towards significant potential biomedical areas, as summarized in Table 2.

## 5. Conclusions

There are many exceptional advantages of photothermal-sensitive GGels in the field of biomedical applications, including NIR-mediated hyperthermic anticancer therapy, NIR-triggered drug release system, antimicrobial and wound healing, tissue engineering and bone regeneration, owing to their fascinating properties such as cost-effectiveness, straightforward functionalization and high photothermal conversion efficiency. GGels could act as innovative materials exhibiting significant potential in biomedical applications. This review provides an overview of GGel synthesis and their unique properties and potential biomedical applications, accelerating the understanding of GGels and attracting the attention of researchers on the fabrication and utilization of photothermal-responsive GGels for photothermal biomedicine. Although GGels-based photothermal biomedicine exhibits highly efficient treatment, there are still some limitations of GGels in phototherapy that prevent them from clinical applications, such as real biotoxicity of GGels in vivo, targeting ability, thermal damage to normal tissues and insufficient therapy effect [156]. Therefore, scientists should develop novel designs that address their issues by enhancing the biocompatibility of GGels, improving targeting ability through specific biomolecules, synergistically employing other nanostructures, combining with other therapeutic approaches. In addition, preclinical studies must be conducted using GGels for photothermal biomedicine to confirm the feasibility of these biomaterials. Despite taking extra time to exploit GGels in clinical trials, GGels that have been continuously explored hold great potential for supporting the feasible applications in photothermal medicine.

## Figures and Tables

**Table 1:** Comparison of preparation techniques for graphene integrated hydrogels.

Fabricated Method	Advantages	Disadvantages
Physically cross-linked fabrication	Forming stable as well as uniform dispersion solution Good electro-conductive, biocompatibility and adsorption ability of heavy metal ions	Limited functional and mechanical properties
Addition of chemical cross-linkers	High water retention ratio Good transparency and self-assembled ability	Poor absorption properties Cross-linking reaction depend on mixing-process
In situ polymerization	Quick and having low energy cost Excellent pH sensitivity and swelling-deswelling ability Strong interfacial interaction	Resistance enhancement upon stretching Readily breakable at low deformation in elongation
Addition of metal ions or hydrophobic monomer	Compositions easily alter via pH adjustment Robust interconnected in 3D networks High toughness, super-stretchability and elasticity	Increased composite’s fracture stress upon GO concentration enhancement

**Table 2:** Polymer component and modification of GGels for different applications of GGels in photothermal biomedicine.

Polymers	Modification	Application	Ref
PAA	-	PTT	[106]
PEGDA	-	PTT	[107]
SF	UCNP	Imaging/PTT	[110]
Glycerol-modified PVA	-	Thermal-triggered drug delivery	[116]
Chitosan	DOX	Thermal-triggered chemotherapy	[117]
poly(N-isopropylacrylamide), poly(2-dimethylaminoethyl) methacrylate)	Sodium alginate and magnetic NPs	Thermal-triggered chemotherapy	[118]
CS, PEG	DOX	PTT/PDT/Chemotherapy	[39]
Polyaspartamide	HA, IT hydrochloride	PTT/Chemotherapy	[125]
CS, Poloxamer 407, Poloxamer 188	Docetaxel	PTT/Chemotherapy	[128]
BPEI	CSMA, DOX	PTT/Chemotherapy	[129]
Agarose, CS	DOX:IBU	PTT/Chemotherapy	[40]
Pluronic F127	DOX	PTT/Chemotherapy	[136]
AE	AuNPs	PTT/PDT	[122]
SE	AuNCs, 5-FU	PTT/PDT/Chemotherapy	[137]
PVP, PAAm	Alg, AuNRs, MTX, RhB	PTT/PDT/Chemotherapy	[123]
Hemin	RhB	Antibacterial	[144]
PVA	MoS_2_/Ag_3_PO_4_	Antibacterial wound healing	[142]
GelMA, HA-DA	βCD, BNN6	Antibacterial wound healing	[143]
HA-DA	HPR	Antibacterial wound healing	[141]
PDA	-	Antibacterial wound healing	[145]
CS	Teriparatide	Tissue repair, bone generation	[149]
nHA	-	Tissue repair, bone generation	[150]
-	BPNFs	Reversible cell capture and on-demand release	[155]
PEGDMA	-	Insulin release	[154]

## Data Availability

No data available.
